# Inhibition of Anchorage-Independent Proliferation and G0/G1 Cell-Cycle Regulation in Human Colorectal Carcinoma Cells by 4,7-Dimethoxy-5-Methyl-l,3-Benzodioxole Isolated from the Fruiting Body of *Antrodia camphorate*

**DOI:** 10.1093/ecam/nep020

**Published:** 2011-06-08

**Authors:** Hsiu-Man Lien, Hsiao-Wei Lin, Ying-Jan Wang, Li-Ching Chen, Ding-Yah Yang, Ya-Yun Lai, Yuan-Soon Ho

**Affiliations:** ^1^Department of Chemistry, Tunghai University, Taichung, Taiwan; ^2^Graduate Institute of Biomedical Technology, Taipei Medical University, Taipei 110, Taiwan; ^3^Department of Environmental and Occupational Health, National Cheng Kung University Medical College, Tainan, Taiwan; ^4^Graduate Institute of Neuroscience, Taipei Medical University, Taipei, Taiwan; ^5^Department of Applied Chemistry, Chung Shan Medical University, Taichung 402, Taiwan

## Abstract

In this study, 4,7-dimethoxy-5-methyl-l,3-benzodioxole (SY-1) was isolated from three different sources of dried fruiting bodies of *Antrodia camphorate* (*AC*). *AC* is a medicinal mushroom that grows on the inner heartwood wall of *Cinnamomum kanehirai Hay* (*Lauraceae*), an endemic species that is used in Chinese medicine for its anti-tumor and immunomodulatory properties. In this study, we demonstrated that SY-1 profoundly decreased the proliferation of human colon cancer cells (COLO 205) through G0/G1 cell-cycle arrest (50–150 **μ**M) and induction of apoptosis (>150 **μ**M). Cell-cycle arrest induced by SY-1 was associated with a significant increase in levels of p53, p21/Cip1 and p27/Kip1, and a decrease in cyclins D1, D3 and A. In contrast, SY-1 treatment did not induce significant changes in G0/G1 phase cell-cycle regulatory proteins in normal human colonic epithelial cells (FHC). The cells were cultured in soft agar to evaluate anchorage-independent colony formation, and we found that the number of transformed colonies was significantly reduced in the SY-1-treated COLO 205 cells. These findings demonstrate for the first time that SY-1 inhibits human colon cancer cell proliferation through inhibition of cell growth and anchorage-independent colony formation in soft agar. However, the detailed mechanisms of these processes remain unclear and will require further investigation.

## 1. Introduction

*Antrodia camphorata* (*A. camphorate, AC*), also called *A. cinnamomea*, is composed of fruiting bodies, mycelium and spores. It is a parasitic fungus that only grows on the inner heartwood wall of *Cinnamomum kanehirai Hay (Lauraceae*). *AC* has been used in traditional Chinese medicine to treat food and drug intoxication, diarrhea, abdominal pain, hypertension, pruritis (skin itch) and liver cancer; however, its biological activities have not been meaningfully investigated to date. Recent studies have demonstrated that *AC* induces significant apoptosis of HL-60 leukemia cells but not of cultured human endothelial cells [1]. Another study demonstrated that *AC* extracts may be used as an adjuvant anti-tumor agent for human hepatoma cells (C3A and PLC/PRF/5), which are resistant to most other anti-tumor agents. Anti-tumor effects were assessed by monitoring tumor growth and the survival rate of xenograft nude mice after combined therapy with several anti-tumor agents [2].

Colon cancer affects 50–60 of every 100 000 people in North America and is the second most common cause of cancer-related death, after lung cancer [3]. The current set of options for treating human colon cancer are limited to surgical resection, general chemotherapy, gene therapy and radiation therapy [4–6]. Therefore, investigators continue to search for new therapeutic strategies such as adjuvant therapies [7]. One approach, as explored in this study, seeks to identify medicinal agents that are capable of arresting the cell cycle and/or activating the cellular apoptotic response in cancerous cells. Our results demonstrate that 4,7-dimethoxy-5-methyl-l,3-benzodioxole (SY-1), isolated from dried fruiting body samples of *AC*, significantly inhibited the proliferation of COLO 205 cells through G0/G1 cell-cycle arrest and apoptosis. We show that SY-1 inhibits the anchorage-independent proliferation of human COLO 205 tumor cells in soft agar colony formation assays. Our results highlight the molecular mechanisms of the anti-tumor effects as mediated by SY-1.

## 2. Materials and Methods

### 2.1. Source of Organism

**Figure 1:**
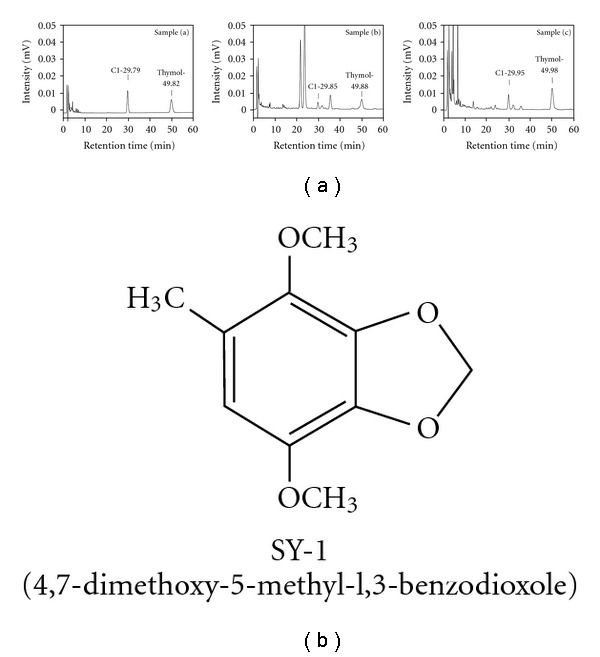
Isolation and chemical structural characterization of *SY-1* from *AC*. (a) HPLC chromatograms of *AC*. Sample a, fruiting bodies of *AC* (YS-187) provided from Yusheng Co., Ltd (spiked concentration 20 mg mL^−1^); sample b, wild fruiting bodies of *AC* from Shinyi, Nantou County in Median Taiwan (spiked concentration 60 mg mL^−1^); and sample c, plate cultivation of fungus *A. camphorota* (BCRC-36795) (spiked concentration 20 mg mL^−1^). (b) Chemical structure of 4,7-dimethoxy-5-methyl-l,3-benzodioxole (SY-1).

As shown in Figure 1(a), fruiting body samples were obtained from three different *AC* sources. Sample a, designated YS-187, was a gift from Yusheng Co., Ltd (Taichung, Taiwan). Sample b was purchased from Shinyi, Nantou County, Taiwan. Sample c, a strain of the fungus *A. camphorota* (BCRC-36795), was purchased from the Food Industry Research and Development Institute (FIRDI), HsinChu County, Taiwan.

### 2.2. Plate Cultivation of *AC*

*AC* (BCRC-36795) was inoculated in a culture medium of potato dextrose agar composed of 0.4% diced potato extract, 2% glucose and 1.5% agar in distilled water. The whole medium was shaken at 28 ± 1°C in darkness, using a 100-rpm rotary shaker for 14 days. The culture broth was collected and evaporated under reduced pressure. The precipitated residue (fruiting body samples of *AC*) was lyophilized at −80°C.

### 2.3. Isolation and Characterization of SY-1 from Fruiting Body Samples of *AC*

The dried fruiting body samples (sample b, BCRC-36795) were ground into a fine powder with an electrical mill and sequentially extracted with ethyl acetate at 80°C under reflux for 3 h. The methanol extracts were separated by gel filtration and chromatographed on a Sephadex LH-20 column (MeOH). The flow rate was 0.5 mL min^−1^. Each fraction measured 15 mL and was collected by a fraction collector. The fractions were further analyzed using a reverse phase C18 column (Mightysil RP-18 GP 250-10, 5 *μ*M). The mobile phase, at a flow rate of 1.5 mL min^−1^, consisted of 2% acetic acid and acetonitrile, programmed as follows: 2% acetic acid–acetonitrile (38 : 62) for 25 min, decreased to 2% acetic acid–acetonitrile (0 : 100) between the 26th and 55th min, then increased again to 2% acetic acid–acetonitrile (38 : 62) between the 56th and 70th min. The spectrophotometric detector was set at 252 nm. Retention fractions were collected at 29.85 min and concentrated by evaporation under reduced pressure. Our results revealed that the yields of SY-1 from dried fruiting body samples a, b and c were 2.5, 0.415 and 5.7 mg g^−1^, respectively. The same fractions were lyophilized to obtain SY-1 (Figure 1(b)). The NMR data of SY-1 is shown as follows: ^1^H NMR (200 MHz, CDCl_3_): **δ** 2.15 (3H, s, CH_3_), 3.82 (3H, s, OCH_3_), 3.85 (3H, s, OCH_3_), 5.91 (2H, s, O–CH_2_–O), 6.27 (1H, s, aromatic H). ^13^C NMR (50 MHz, CDCl_3_): **δ** 15.92 (CH_3_); 56.85 (OCH_3_); 59.94 (OCH_3_); 101.44 (O–CH_2_–O); 108.72; 123.66; 134.63; 136.51; 138.64; 138.83. MS (M^+^) *m/z* 196. Anal. (C_10_H_12_O_4_) C, H.

### 2.4. Cell Lines, Cell Culture and Cell Growth Curves

The HT 29 (p53 His^273^mutant) [8] and COLO 205 (p53 wild type) [9] cell lines were isolated from human colon adenocarcinomas (HSY-1-38 and CCL-222; American Type Culture Collection). The Hep G2 (p53 wild type, ATCC HB-8065) and MDA-MB-231 (mutant p53, ATCC HTB 26) cell lines were derived from a human hepatocellular carcinoma and human mammary gland epithelial adenocarcinoma, respectively [9–12]. The oral squamous cell carcinoma cell line (Ca9-22), bearing a mutant p53 gene, was used as a research model [13]. FHC (CRL-1831; American Type Culture Collection) is a cell line derived from long-term epithelial cell cultures of normal human fetal colonic mucosa [14]. A total of 1 × 10^4^ cells were seeded in a 35 mm Petri dish and treated with SY-1 (75–375 *μ*M) for cell growth proliferation assays.

### 2.5. Determination of Cell Viability

After treatment with SY-1, cell growth curves were determined at the indicated time points with the 3-(4,5-dimethylthiazol-2-yl)-2,5-diphenyl-2H-tetrazolium bromide (MTT) assay.

### 2.6. Flow Cytometry

COLO 205 cells were synchronized as previously described [15]. The cells were harvested at various times with trypsin-EDTA, washed twice with PBS/0.1% dextrose, and fixed in 70% ethanol at 4°C. Nuclear DNA was stained with a reagent containing propidium iodide (50 *μ*g mL^−1^) and DNase-free RNase (2 U mL^−1^) and measured by a fluorescence-activated cell sorter (FACS). The population of nuclei in each phase of the cell cycle was determined using the well-known CellFIT DNA analysis software (Becton Dickenson, San Jose, CA).

### 2.7. Protein Extraction and Western Blot Analysis

Western blot analysis was performed as previously described [15, 16]. Immunodetection was carried out by probing with appropriate dilutions of specific antibodies at room temperature for 2 h. Anti-p21/Cip1, anti-p27/Kip1, anti-p53, anti-GAPDH monoclonal antibodies (Santa Cruz, Inc., CA, USA) and anti-cyclin D1, anti-cyclin D3, anti-CDK2, anti-CDK4 and anti-cdc 25C monoclonal antibodies (Transduction Laboratories, Lexington, KY) were used at a dilution of 1 : 1000. Anti-cyclin A polyclonal antibodies (Transduction, San Diego, CA) were used at a dilution of 1 : 250. The secondary antibodies, alkaline phosphatase-coupled anti-mouse and anti-rabbit antibody (Jackson, Westgrove, PA), were incubated at room temperature for 1 h at dilutions of 1 : 5000 and 1 : 1000, respectively.

### 2.8. Analysis of Apoptosis

Apoptosis in COLO 205, HT 29 and FCH cells subjected to the various treatments was determined by DNA fragmentation analysis [17]. Genomic DNA was quantified, and equal amounts of DNA sample were electrophoresed in a 2% agarose gel. The DNA was visualized using ethidium bromide staining.

### 2.9. Soft Agar Cloning Assay

The base layer consisted of 0.9% low-gelling point SeaPlaque agarose (Sigma, St Louis, MO) in complete COLO 205 culture medium. Soft agar consisting of 0.4% SeaPlaque agarose in complete COLO 205 culture medium was mixed with 1 × 10^4^ COLO 205 cells and plated on top of the base layer in a culture dish of 60 mm diameter. Soft agar cultures were maintained at 37°C and observed with a Leica DMI 4000B Microscope Imaging System to evaluate colony counts.

### 2.10. Statistics

All data are reported as means ± SE. Comparisons were subjected to one-way analysis of variance (ANOVA) followed by Fisher's least significant difference test. Significance was defined as *P* <  .05.

## 3. Results

### 3.1. Isolation of SY-1 from *AC* and Its Structural Characterization

As seen in Figure 1(a), three different sources of *AC* were isolated via extensive chromatographic purification of the ethyl acetate-soluble fraction of the dried fruiting body (Figure 1(a), samples a–c). One major peak was seen for each sample (indicated as compound 1, C1). The chemical structure of the purified white powder (C1) was elucidated by NMR spectroscopy and mass spectrometry studies and was identified as SY-1 (Figure 1(b)).

### 3.2. Inhibition of Malignant Human Colon Cell Proliferation by SY-1-Treatment

**Figure 2:**
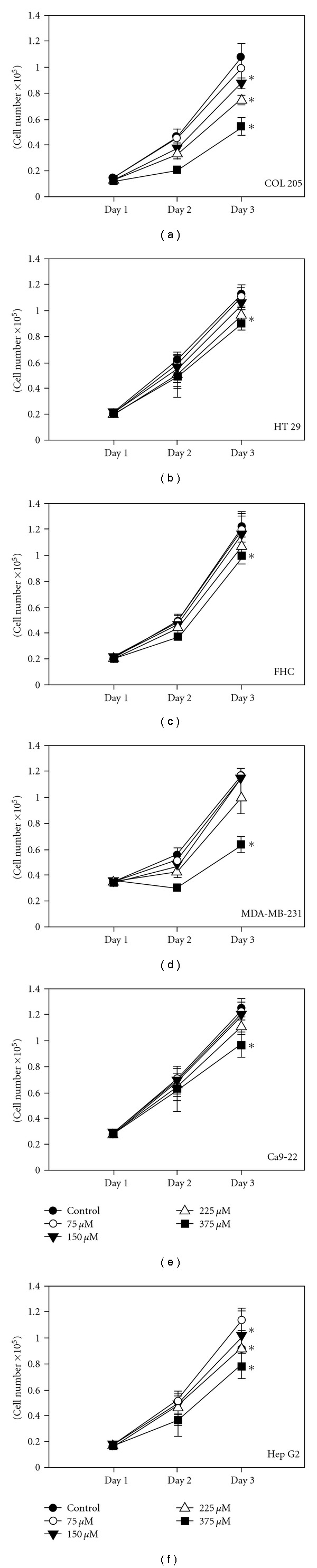
Dose-dependent effects of SY-1 on cell growth in malignant and normal human cells. (a) COLO 205, (b) HT 29, (c) normal human colonic epithelial (FHC), (d) MDA-MB-231, (e) Ca9-22 and (f) Hep G2 cells were treated with various concentrations of SY-1 (75–375 *μ*M). Media with or without SY-1 was renewed daily prior to cell counting. Three samples were analyzed in each group. Values represent mean ± SE.

We examined the effect of SY-1 on the growth of human cancer cells with various p53 statuses and on the growth of normal human colonic epithelial cells. The cells were cultured for 3 days with or without SY-1 (75–375 *μ*M) and were then harvested and counted. Our results suggest that SY-1 (>150 *μ*M *P* < 5) significantly inhibited cell growth in COLO 205 and HepG2 cells in a dose- and time-dependent manner (Figures 2(a) and 2(f)). However, inhibition of the cancer lines HT-29, MDA-MB-231 and Ca9-22 as well as normal (FHC) cells only occurred at higher concentrations of SY-1 (>375 *μ*M) (Figures 2(b), 2(c), 2(d) and 2(e)). These results suggest that the wild-type p53 cancer cells were more sensitive to SY-1 treatment than cancer cells with mutated p53 were.

**Figure 3:**
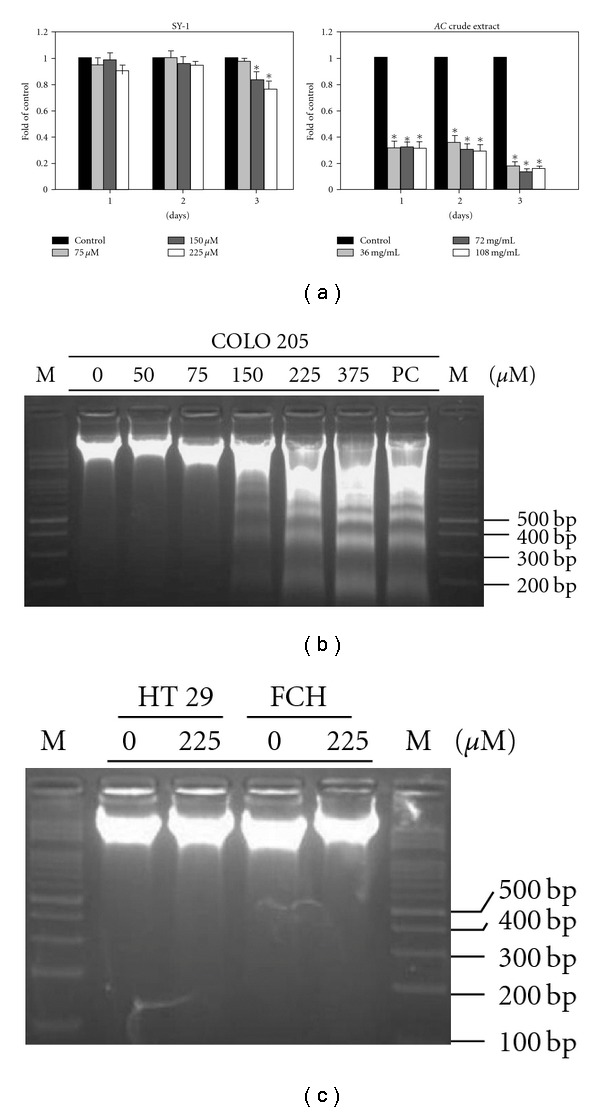
Dose-dependent SY-1-induced DNA fragmentation in human colon cancer and normal cells. (a) COLO 205 cells were treated with SY-1 and *AC* crude extract in a dose- and time-dependent manner. (b) COLO 205, (c) HT 29 and normal human colonic epithelial (FHC) cells were treated with SY-1 at the indicated doses. Induction of apoptosis in all cells was shown by DNA fragmentation using electrophoresis of genomic DNA. DNA fragmentation was examined 24 h after drug treatment.

The yield of SY-1 from dried fruiting body samples (sample b, BCRC-36795) was 0.415 mg g^−1^. To evaluate the anti-proliferative effects of SY-1, isolated from crude extracts of *AC*, an equal concentration of SY-1 from *AC* extracts was applied to COLO 205 cells and the cytotoxicity assay was performed (Figure 3(a)). A concentration of greater than 150 *μ*M SY-1 significantly induced cytotoxicity in COLO 205 cells, but lower concentrations had no cytotoxic effects (Figure 3(a), upper panel). However, significant cytotoxic effects was observed in COLO 205 cells treated with 36 mg mL^−1^ of *AC* crude extract which contained an identical concentration of 75 *μ*M SY-1 (Figure 3(a), lower panel). We next investigated whether the cytotoxic effect of SY-1 on COLO 205 cells was due to apoptotic cell death. SY-1-induced apoptosis was evaluated by DNA fragmentation analysis. As shown in Figure 3(b), DNA fragmentation was only observed in the SY-1-treated COLO 205 cells (>150 *μ*M). In contrast, DNA fragmentation induced by a higher dose of SY-1 (225 *μ*M) was not observed in either the HT 29 or FCH cells (Figure 3(c)). These results suggest that SY-1-induced apoptosis, as observed in the COLO 205 cells, occurs in a cell-specific manner.

### 3.3. Arrest of Cell Cycle at the G0/G1 Phase by SY-1 in Human COLO 205 Cells

**Figure 4:**
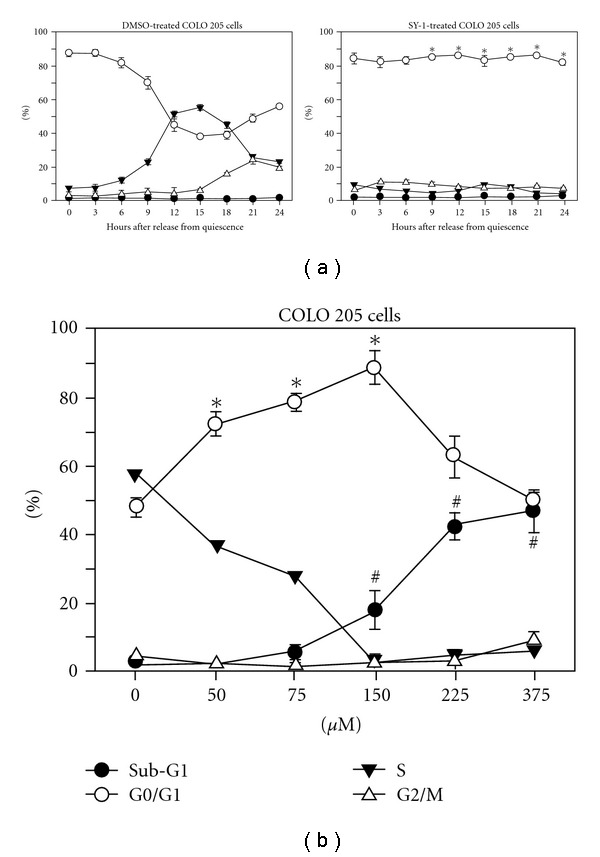
Time- and dose-dependent SY-1-induced G0/G1 phase arrest and apoptosis in COLO 205 cells. (a) COLO 205 cells were synchronized with 0.04% FCS for 24 h as described in “Materials and Methods” section. After synchronization, the cells were released into complete medium (10% FCS) containing 0.05% DMSO (left panel), or 150 *μ*M SY-1 in 0.05% DMSO (right panel). The percentages of cells in the Sub-G1, G0/G1, S and G2/M phases of the cell cycle were determined using the well-known CellFIT DNA analysis software. Three samples were analyzed in each group, and values represent the mean ± SE (b). Dose-dependent effects of SY-1 on cell cycle and apoptosis in human cancer cells. FACS analysis of DNA content 15 h after release from quiescence by incubation in culture media supplemented with 10% FCS and various concentrations of SY-1 in 0.05% DMSO. The percentage of cells in the Sub-G1, G0/G1, S and G2/M phases of the cell cycle were determined using the CellFIT DNA analysis software. Three samples were analyzed in each group, and values represent the mean ± SE.

As shown in Figure 2(a), concentrations of SY-1 ranging from 75 to 350 *μ*M induced a dose-dependent inhibition of cell growth in human COLO 205 cancer cells. We further demonstrated significant apoptosis at doses greater than 150 *μ*M (Figure 3(b)). These results imply that lower doses of SY-1 (<150 *μ*M) should affect the cell cycle. To more clearly demonstrate the actions of SY-1 during a specific phase of the cell cycle, the COLO 205 cancer cells were synchronized by switching them to media that contained 0.04% FCS for 24 h to render them quiescent [15]. Subsequently, the cells were returned to culture media containing 10% FCS and 0.05% DMSO with or without 150 *μ*M SY-1. The cells were harvested for flow cytometry analysis of DNA content at various times thereafter (Figure 4(a)). The results demonstrate that SY-1 induced an accumulation of greater than 85% of COLO 205 cells at the G0/G1 phase of the cell cycle, suggesting that the observed growth inhibitory effect of SY-1 was due to an arrest at the G0/G1 phase of the cell cycle.

Figure 4(b) demonstrates the dose effects of SY-1 on G0/G1 arrest. According to our previous studies [18, 19] and the representative FACS analysis (Figure 4(a), left panel), 0, 15, 21 and 24 h represent the G0/G1, S, G2/M and 2nd G0/G1 phases, respectively. The greatest difference in the G0/G1 cell populations of the SY-1-treated and control groups was recorded at 15 h after replacement with complete medium. Accordingly, this time point was selected to study the dose-dependent effect of SY-1 and to identify the minimal dose of SY-1 required for induction of G0/G1 arrest, as determined by flow cytometry analysis. As illustrated in Figure 4(b), significant G0/G1 arrest in COLO 205 cells was induced by treatment with SY-1 (>50 *μ*M) in a dose-dependent manner. We further demonstrated that the Sub-G1 (apoptotic) population of COLO 205 cells was significantly increased by exposure to more than 150 *μ*M SY-1 for 15 h.

### 3.4. The Effects of SY-1 on G0/G1 Phase Cell-Cycle Regulatory Proteins

To investigate the underlying molecular mechanisms of SY-1-induced G0/G1 arrest, the COLO 205 cells were switched to media with 0.04% FCS to render them quiescent at the G0/G1 phase. They were then returned to culture media supplemented with 10% FCS and 0.05% DMSO with or without SY-1 (75–225 *μ*M). After 15 h they were harvested for protein extraction and western blot analysis to examine the effects of SY-1 on the expression of G0/G1 phase cell-cycle regulatory proteins.

**Figure 5:**
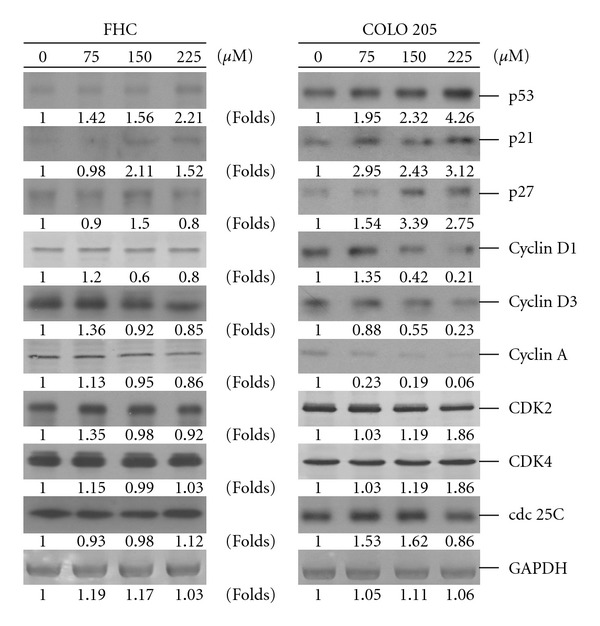
Dose effect of SY-1 on the concentrations of cell-cycle regulatory proteins. Normal human colonic epithelial (FHC, left panel) and cancer (COLO 205, right panel) cells were rendered quiescent for 24 h and then given 10% FCS in the presence or absence of SY-1 (75–150 *μ*M) for an additional 15 h. Protein extracts (100 *μ*g/lane) were separated by SDS-PAGE, probed with specific antibodies and detected using the nitro blue tetrazolium and 5-bromo-4-chloro-3-indolyl-phosphate systems. Membranes were also probed with an anti-GAPDH antibody to correct for differences in protein loading.

Recent studies have demonstrated that the cyclin-dependent kinase (CDK) inhibitors, including p21/Cip1 and p27/Kip1, are up-regulated in human cancer cells arrested in G0/G1 by treatment with anti-cancer agents [20, 21]. Our previous study showed that the protein levels of p21/Cip1 and p27/Kip1 were increased in the quiescent COLO 205 cells and then decreased 15 h after release from quiescence [22]. Similar results were observed in our present study. The protein levels of p21/Cip1 and p27/Kip1 were lower at 15 h after the addition of 10% FCS medium (Figure 5, right panel, lane 1). In contrast, increased p21/Cip1 and p27/Kip1 protein levels were induced in the SY-1 (>70 *μ*M)-treated COLO 205 cells (Figure 5, right panel, lanes 2–4). The levels of cyclins A, D1 and D3 were down-regulated in the SY-1-treated cells, while the levels of CDK2 and CDK4 proteins did not change (Figure 5, right panel, lanes 2–4). Cdc 25C, which promotes cell entry into the S and the G2/M phases, was also down-regulated at a higher SY-1 dose (>225 *μ*M) (Figure 5, right panel, lane 4). As a normal cell control, human FCH cells were treated with SY-1 at the same concentrations. Our results show that the cell-cycle regulatory proteins of FCH cells were substantially less affected than the SY-1-treated group (Figure 5, left panel).

### 3.5. P53-Activated Signaling Pathway Is Involved in SY-1-Induced G0/G1 Arrest

Our previous results show that significant G0/G1 arrest (50–150 *μ*M) and apoptosis (above 150 *μ*M) were induced by SY-1 in human COLO 205 cancer cells in the context of wild-type p53 (Figures 3 and 4). In contrast, SY-1-induced apoptosis was not observed in the HT 29 (p53 His^273^ mutant) cells (Figure 3(c), lane 2). The p53 protein is a potent transcription factor that is involved in the regulation of cell-cycle arrest and in the induction of apoptosis [23, 24]. The data here suggest that the ability of SY-1 to induce human cancer cells to undergo G0/G1 cell-cycle arrest or apoptosis is dependent on the p53 status of the cells. Interestingly, the level of p53 protein remained unchanged in the SY-1-treated human colonic epithelial (FHC) cells (Figure 5). Such observations can explain why the SY-1-induced antiproliferative effects were tumor cell specific (Figure 1). In summary, our results demonstrate that the levels of p53 and its downstream regulation of p21/Cip1 and p27/Kip1 proteins were dose-dependently increased in the SY-1-treated COLO 205 cells. This suggests that upregulation of p53 and p21/Cip1 might be involved in SY-1-mediated arrest in these cells.

**Figure 6:**
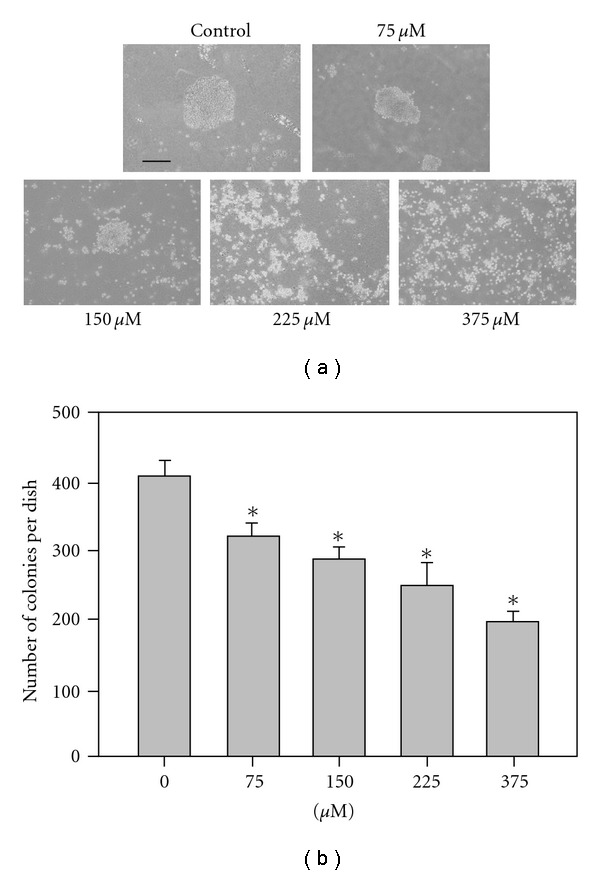
Anchorage-independent growth of SY-1-treated COLO 205 cells in soft-agar. (a) The COLO 205 cells were treated with SY-1 (75 *μ*M) following methods described elsewhere [26]. The gross morphology of COLO 205 cell colonies are shown here on culture plates. These colonies exhibit subtle changes in their morphology, including a slight disaggregation compared to the untreated COLO 205 cells. Bar = 200 *μ*M. (b) The number of colonies scored from the soft-agar plates. The COLO 205 cells were seeded in soft agar with or without SY-1 (75–375 *μ*M). The colonies were counted in a 1 × 3 cm^2^ area on each plate. Data are the mean ± SE of three different experiments. Significance was accepted at *P* < .05. Asterisk indicates that the SY-1-treated groups were significantly different from the DMSO-treated group.

In contrast to normal adherent cells, tumor cells have the ability to grow without binding to a substrate. Our data suggests that anchorage-independent proliferation is a hallmark of tumor cell malignancy [25]. The anchorage-independent proliferation of COLO 205 cells was investigated in soft agar colony formation assays (Figure 6). Twelve days after plating, both the control and SY-1-treated COLO 205 cells showed colony formation in medium containing 0.3% agar. As shown in Figure 6(a), the average sizes of the control COLO 205 colonies were 2-3 times larger than the SY-1-treated group. Compared to the DMSO-treated control cells, SY-1 (>75 *μ*M) significantly reduced the number of colonies formed in a dose-dependent manner (Figure 6(b), lanes 2–5).

## 4. Discussion

**Figure 7:**
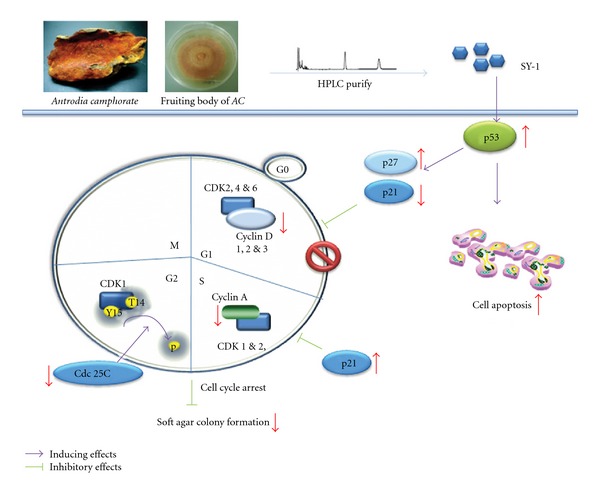
Schematic diagram of signaling pathways involved in SY-1-induced cell-cycle arrest and apoptosis in human COLO 205 cells. SY-1 was isolated from the fruiting body of *AC* (upper left). After treatment with SY-1, the p53-regulated p21/p27 signals in COLO 205 cells resulted in inhibition of soft agar colony formation.

The present study was undertaken to investigate the anti-cancer mechanisms of SY-1 isolated from three different sources of dried fruiting bodies from the Chinese medicinal mushroom, *Antrodia camphorate* (Figure 7). Previous studies have demonstrated that *AC* induces significant apoptotic cell death in human leukemia (HL-60) [27], breast (MCF-7 and MDA-MB-231) [27, 28], prostate (LNCaP and PC3) [29] and liver (Hep G2, C3A and PLC/PRF/5) cancer cells [2, 30]. Such an effect has never been detected with cultured human endothelial cells [1]. The anti-proliferative effects of *AC* were also reported in different types of human cancer cells including breast [27, 28], prostate [29], bladder [31], lung [32] and hepatoma [2]. These results demonstrate that both cell-cycle inhibition and apoptotic cell death contribute to the anti-tumor effects of *AC*. In a previous paper [33], three purified compounds were isolated from *AC*, namely sesquiterpene lactone (antrocin), SY-1 and 2,2′,5,5′-tetramethoxy-3,4,3′4′-bimethylenedioxy-6,6′-dimethylbiphenyl. To our knowledge, ours is the first demonstration that a purified compound (SY-1), isolated from *AC*, inhibits the cell growth of COLO 205 cells through arrest of the cell cycle and activation of the cellular apoptotic response.

In this study, the inhibitory effect of SY-1 on cell growth appears to be limited to COLO 205 and Hep G2 cells, since similar effects were not observed in other transformed colon cancer (HT 29) cells (Figure 2). Treatment of COLO 205 cells with SY-1 resulted in an increase in the levels of p21/Cip1, p27/Kip1 and p53 proteins, and a decrease in the levels of cyclins A, D1 and D3 (Figure 5). Among these changes, p53 seems to have a major role in SY-1-induced G0/G1 arrest in COLO 205 cells (Figure 7). It seems that SY-1 exerts its anti-tumor activity through cell-cycle arrest or activation of the cellular apoptotic response, depending on the p53 status. A previous report demonstrated that HT-29 cells contain a point mutation at codon 273 (Arg→His) of the *p53* gene [8], making it a less efficient inducer of apoptosis [34]. On the other hand, over-expression of mutant p53 is a common theme in human tumors, suggesting a tumor-promoting gain-of-function associated with mutant p53 [35]. A recent study demonstrated that knockdown of mutant p53 sensitizes human colon tumor cells to growth suppression under the activity of various chemotherapeutic drugs [35]. Such results suggest that COLO 205 cells that contain wild-type p53 are susceptible to SY-1-mediated anti-tumor effects. Moreover, SY-1-induced G0/G1 cell-cycle regulatory proteins did not change significantly in the normal colonic cells, namely FHC (Figure 5, left panel). Additional signals, other than p53, may be involved in COLO 205, but are not involved with FHC cells.

Human colonic cancer cells often respond in only a limited manner to the currently available chemotherapeutics due to the expression of multidrug resistance genes [36]. Indeed, human cancer cells with mutant p53 seem to activate the promoters of genes that are not usually activated by wild-type p53 protein, such as multidrug resistance gene 1 (MDR1) and c-MYC [37]. Such results suggest that combination therapy is one potential strategy for reducing a compound's undesirable toxic effect while still maintaining or enhancing its anti-tumor efficacy. In the present study, our results demonstrate that SY-1 inhibits colonic cancer cell growth in the G0/G1 phase, induces apoptosis and inhibits colony formation in COLO 205, but not in HT 29 cells. Recent epidemiologic and laboratory investigations suggest that aspirin and other nonsteroidal anti-inflammatory drugs (NSAIDs) exhibit chemopreventive effects against colon cancer, perhaps due, at least in part, to their activity against cyclooxygenase-2 (COX-2) [38]. In addition, induction of apoptosis by *AC*, through inhibition of COX-2, has also been reported in human breast cancer cells (MDA-MB-231) [27]. *In vivo* studies have demonstrated that *AC* extract treatment of human hepatoma cells (C3A and PLC/PRF/5) inhibits MDR gene expression and the pathway of COX-2-dependent inhibition of p-AKT to induce apoptosis [2]. To our knowledge, COX-2 is strongly expressed in all metastatic cell lines (HT-29) but not in non-metastatic lines (COLO 205) [39]. These results imply that *AC*-mediated COX-2 inhibition is important for inhibition of colon cancer cell growth. However, our results demonstrate that inhibition of cell growth in the HT 29 cells was less profound than in COLO 205 cells (Figure 2), indicating that SY-1, although isolated from *AC*, may not exert its anti-tumor effects completely through the inhibition of COX-2. In summary, although animal studies of SY-1-induced anti-tumor activity are still ongoing, the findings from the present *in vitro* study suggest the potential applications of SY-1 in the treatment of human cancer. The universality of SY-1 in the inhibition of cancer cell proliferation makes it an attractive agent for chemotherapy.

## Funding

National Science Council of R.O.C. (NSC 96-2628-B-038-003-MY3, NSC 95-2320-B-038-016-MY3 to Y.-S.H. and NSC 96-2320-B-040-010 to Y.-Y.L.).
